# Resistance fracture of minimally prepared endocrowns made by three types of restorative materials: a 3D finite element analysis

**DOI:** 10.1007/s10856-021-06610-x

**Published:** 2021-10-30

**Authors:** Qingzhen Meng, Yuejiao Zhang, Danlu Chi, Qimei Gong, Zhongchun Tong

**Affiliations:** 1 Department of Operative Dentistry and Endodontics, Guanghua School of Stomatology, Sun Yat-sen University, Guangzhou, Guangdong China; 2 Guangdong Provincial Key Laboratory of Stomatology, Sun Yat-sen University, Guangzhou, Guangdong China; 3 The First Affiliated Hospital, Sun Yat-sen University, Guangzhou, Guangdong China

**Keywords:** Endocrown, Finite element analysis (FEA), Maximum principal stress (MaxPS), Minimum principal stress (MinPS)

## Abstract

A thin endocrown restoration was often applied in endodontically treated teeth with vertical bite height loss or inadequate clinical crown length. A model of mandibular molars made by endocrown restoration with 1 mm thickness and 2 mm depth of pulp chamber was constructed and imported into FEA ANSYS v18.0 software. The three CAD/CAM materials, feldspathic (Mark2), lithium disilicate (EMAX), and lava ultimate (LU), were assigned, and the five load indenters were loaded on the full occlusal (FO), occlusal center (OC), central fossa (CF), buccal groove (BG), and mesiobuccal cusp (MC) of restoration in the model. The MinPS and MaxPS of the thin endocrown were significantly higher than those of tooth tissue in five types of loads except for the LU endocrown loaded in the FO group. The smaller the contact surface of the load was, the higher MaxPS and MinPS were. MaxPS and MinPS of the MC were the highest, followed by the BG and CF in the restoration. In the stress distribution of tooth tissue, MaxPS in the LU endocrown accumulated at the external edge of enamel and was significantly higher than MaxPS in Mark2 and EMAX endocrown concentrated on the chamber wall of dentin under OC, CF and BG loads. Within the limitations of this FEA study, the LU endocrown transferred more stress to tooth tissue than Mark2 and EMAX, and the maximum principal stress on endocrown restoration and tooth tissue at the mesiobuccal cusp load was higher than that at the central fossa and buccal groove load.

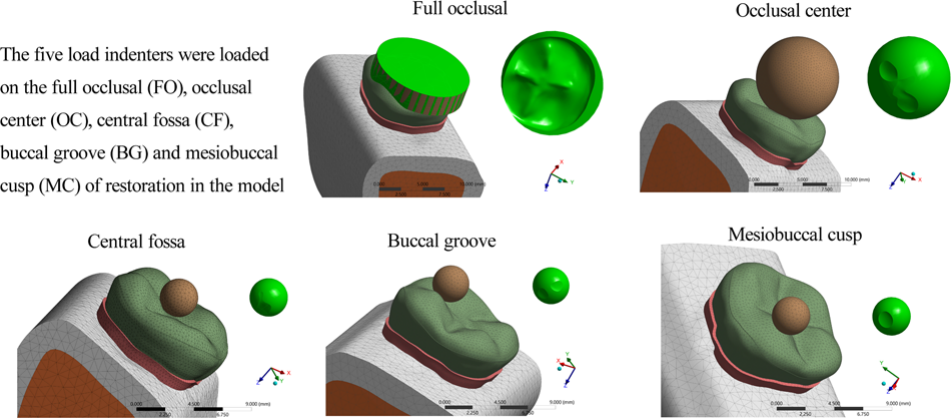

## Introduction

The restoration of endodontically treated teeth with extensive damage remains a clinical challenge [1]. Despite the clinical success obtained by the use of intraradicular posts and full-coverage crowns, one disadvantage of this method is the extra removal of sound tissue needed for fitting the post-retained foundation restoration [2]. Endocrown, a monoblock restoration, assembles the intraradicular post, the core and the crown in one component and was first introduced as an alternative treatment modality in 1995 by Pissis [3]. Endocrown is a reliable alternative to post-retained restorations and has often been used to restore endodontically treated teeth with significant loss of coronal tooth structure [4, 5].

A conservative coronal restoration endocrown utilizes the pulp chamber and adhesive cementation to increase the macromechanical retention of the restoration and reduces the removal of sound tooth tissue [6, 7]. The preservation of more sound tooth tissue signifies a thinner coronal restoration. As with any other restoration, an overall reduction of at least 2 mm in height is required in tooth preparation for an endocrown [8]. Along with the evolution of high-strength and tough restorative materials, a lower occlusal reduction was recommended to adopt a minimally invasive approach in the posterior region [9]. Etchable glass ceramic and nanocomposite resin materials are often used in chairside CAD/CAM fabrication of restorations due to their esthetic appearance, excellent biocompatibility, and mechanical properties [10–12]. Among the available chairside CAD/CAM materials, lithium disilicate glass–ceramic and nanofilled composite resin stand out [13]. Resin nanoceramics (Lava Ultimate, LU) display high-impact fracture resistance, low modulus and high flexural strength, and lithium disilicate (IPS e.max CAD, Ivoclar Vivadent, Schaan, Liechtenstein) possess excellent biomechanical characteristics with a superior flexural strength beyond 400 MPa [11, 12]. Therefore, LU and EMAX are often studied as thinner occlusal veneers to restore the occlusal appearance of defective teeth [14, 15].

Restoration thickness determines the fracture resistance of full-coverage occlusal veneers [14, 16]. Endodontically treated teeth with vertical bite height loss or inadequate clinical crown length are not uncommon and are difficult to rehabilitate using a complete crown; thus, an endocrown may be an optional selection. Under such circumstances, the restoration thickness of the endocrown did not reach at least a 2-mm requirement. Furthermore, from minimally invasive dentistry, a thinner occlusal thickness of endocrown was recommended due to the lower amount of sound tooth tissue removed. Therefore, we need to investigate the stress distribution of the endocrown with a thinner coronal to expect clinical application. In this study, finite element analysis was used to evaluate the stress distribution where the three types of CAD/CAM dental material of endocrown restoration had 1 mm of coronal thickness by the five types of load.

## Methods and materials

### The finite element analysis model

A 3D geometric model of an intact mandibular molar was obtained from microcomputed tomography (uCT50, Switzerland) with a voxel dimension of 9 μm and was reconstructed using a CAD software program and a reverse engineering program (Mimics Medical 20.0; Materialize NV and Geomagic Studio 12.0; Geomagic Inc). The constructed model simulates the endocrown restoration of a mandibular molar after root canal treatment. The external and internal contours of the tooth, alveolar bone (cortical and spongious bone), 0.3-mm-thick periodontal ligament, and dentin and pulp contours were outlined and assembled. A 3-D numerical model of the intact mandibular molar was constructed by assembling all the individual elements. In the model of the tooth with an endocrown restoration, the pulp in the root canal was replaced by gutta-percha, and the traditional endodontic cavity was filled with smart dentin resin (SDR) and the endocrown restoration. The endocrown restoration was designed with 1 mm of occlusal clearance and 2 mm of depth in the pulp chamber, and the axial walls presented an internal taper of 6°. The mechanical properties of the materials, tooth tissue, and bone (elastic modulus and Poisson ratio) were determined from published values, and three CAD/CAM restorative materials were simulated: feldspathic (Mark2), lithium disilicate (EMAX), and lava ultimate (LU) (Table 1). The luting cement between the endocrown restoration and the tooth was limited to 100-μm thickness. The Mark2 and EMAX restorations were adhered using Multilink N, and the LU restoration was adhered with RelyX™ Ultimate. The model was imported into finite element analysis software (FEA, ANSYS, v18.0; Swanson Analysis Inc.). All structures were assumed to be linearly elastic, isotropic, and homogeneously distributed. Nodal displacements on the surfaces of the model were constrained in all directions.

**Table 1:** Material properties [11, 30–33]

Material	Elastic modulus (MPa)	Poisson ratio
Enamel	84,100	0.33
Dentin	18,600	0.31
Periodontal ligament	68.9	0.45
Cortical bone	13,700	0.30
Spongious bone	1370	0.30
Gutta-percha	140	0.45
SDR	12,600	0.24
Lava ultimate	12,700	0.45
IPS e.max CAD	102,700	0.22
Mark2	71,300	0.23
RelyX™ ultimate	7700	0.30
Multilink N	7000	0.30
Apple pulp	3410	0.10
Bone fragment	13,700	0.30

### Stress analysis of the different loads

**Fig. 1:**
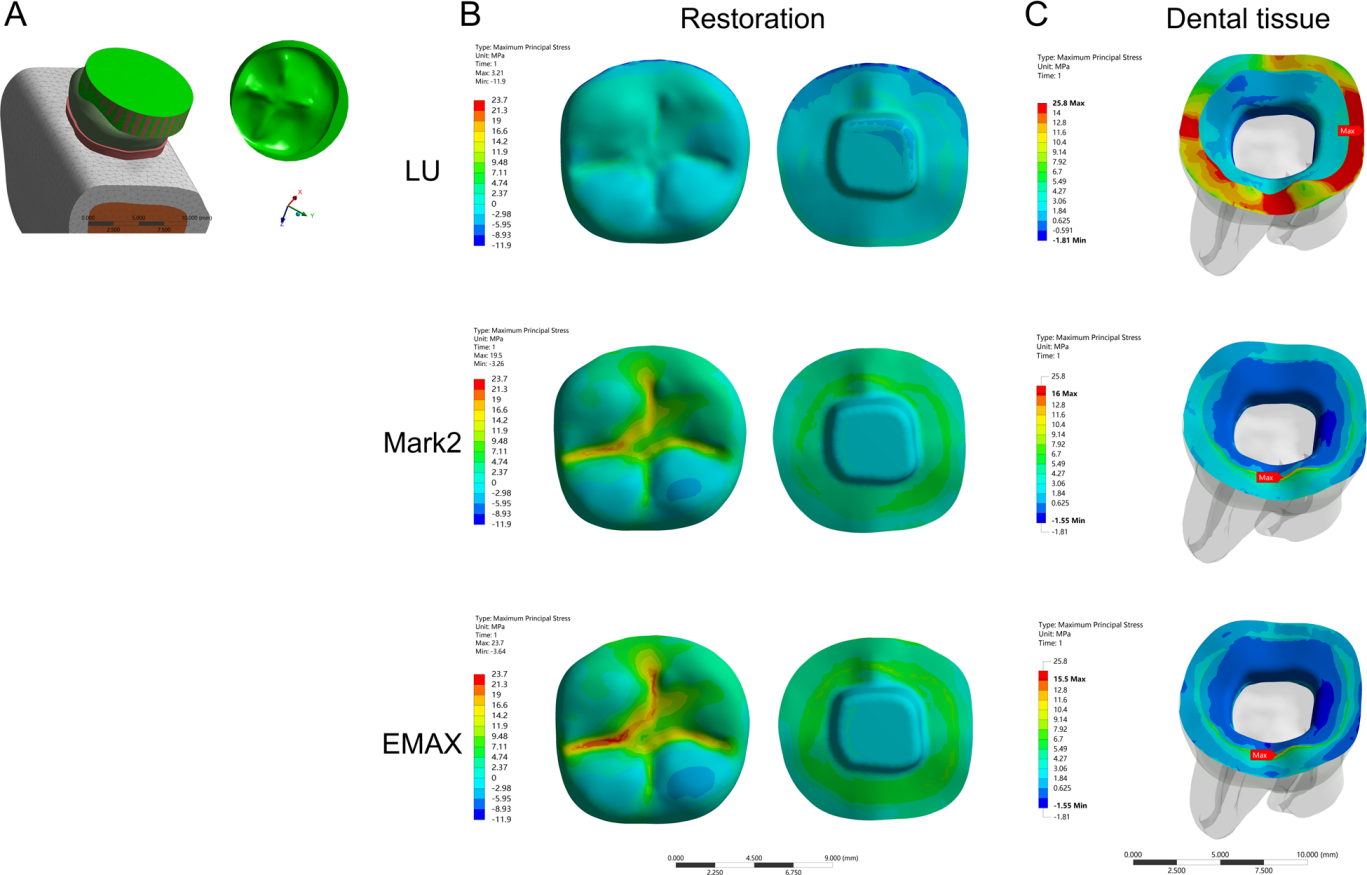
Stress distribution of a mandibular molar covered by an endocrown restoration with a 1-mm thickness and a 2-mm depth of pulp chamber. A cylinder indenter loaded the full occlusal (FO) surface of the restoration except for the lingual edge (contact area: 108.57 mm^2^) to simulate central occlusion (**A**). The restorations were made with lava ultimate (LU), feldspathic (Mark2) and lithium disilicate (EMAX). The MaxPS and MinPS values of the restoration (**B**) and tooth tissue (**C**) are displayed

**Fig. 2:**
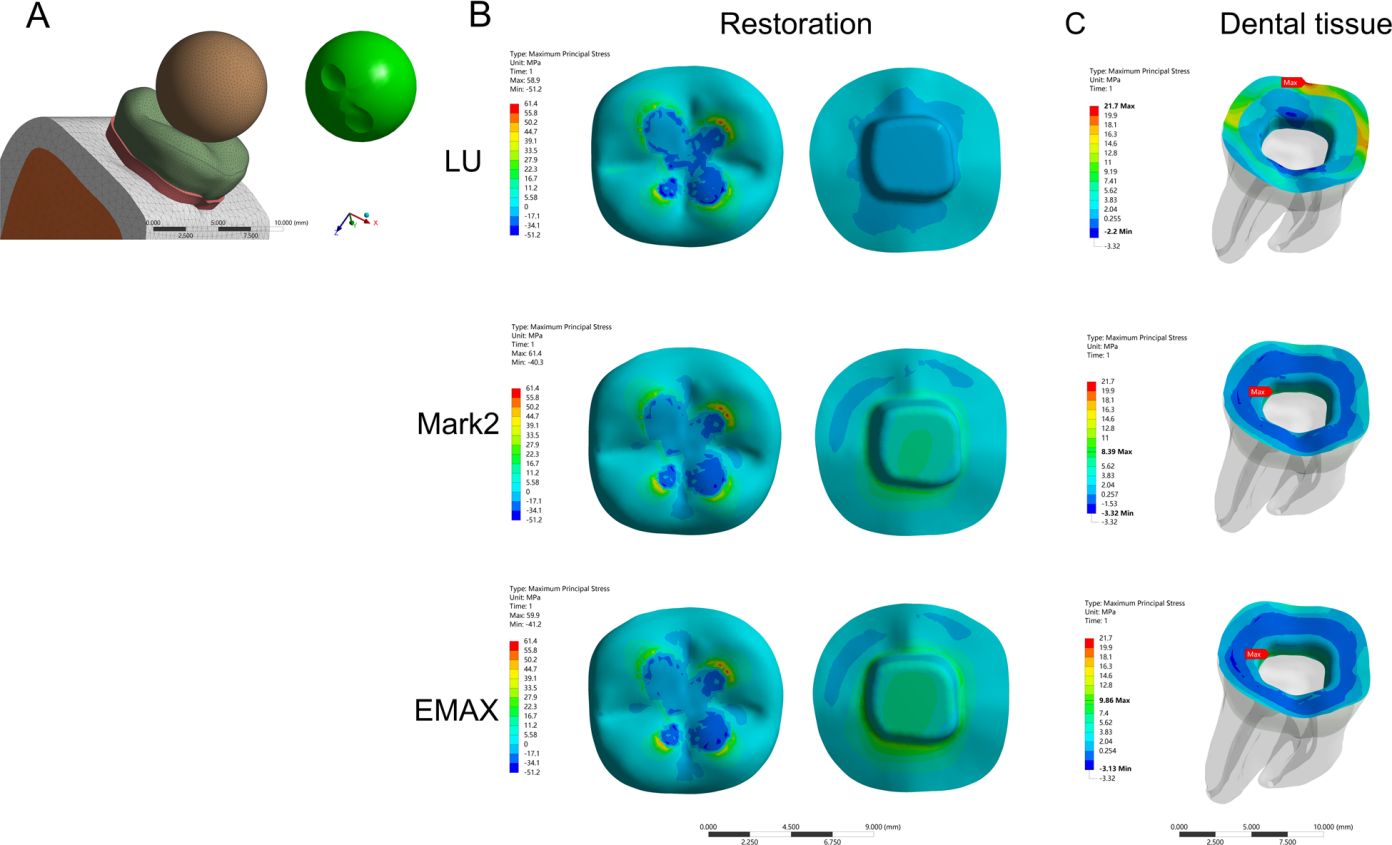
Stress distribution of the mandibular molar covered by an endocrown restoration with a 1-mm thickness and a 2-mm depth of pulp chamber. A large spherical indenter (diameter: 8.57 mm) was generated by revolving in ANSYS v18.0 software and contacting the occlusal center (OC) area of the endocrown restoration (11.425 mm^2^) (**A**). The restorations were made with lava ultimate (LU), feldspathic (Mark2) and lithium disilicate (EMAX). The MaxPS and MinPS values of the restoration (**B**) and tooth tissue (**C**) are displayed

**Fig. 3:**
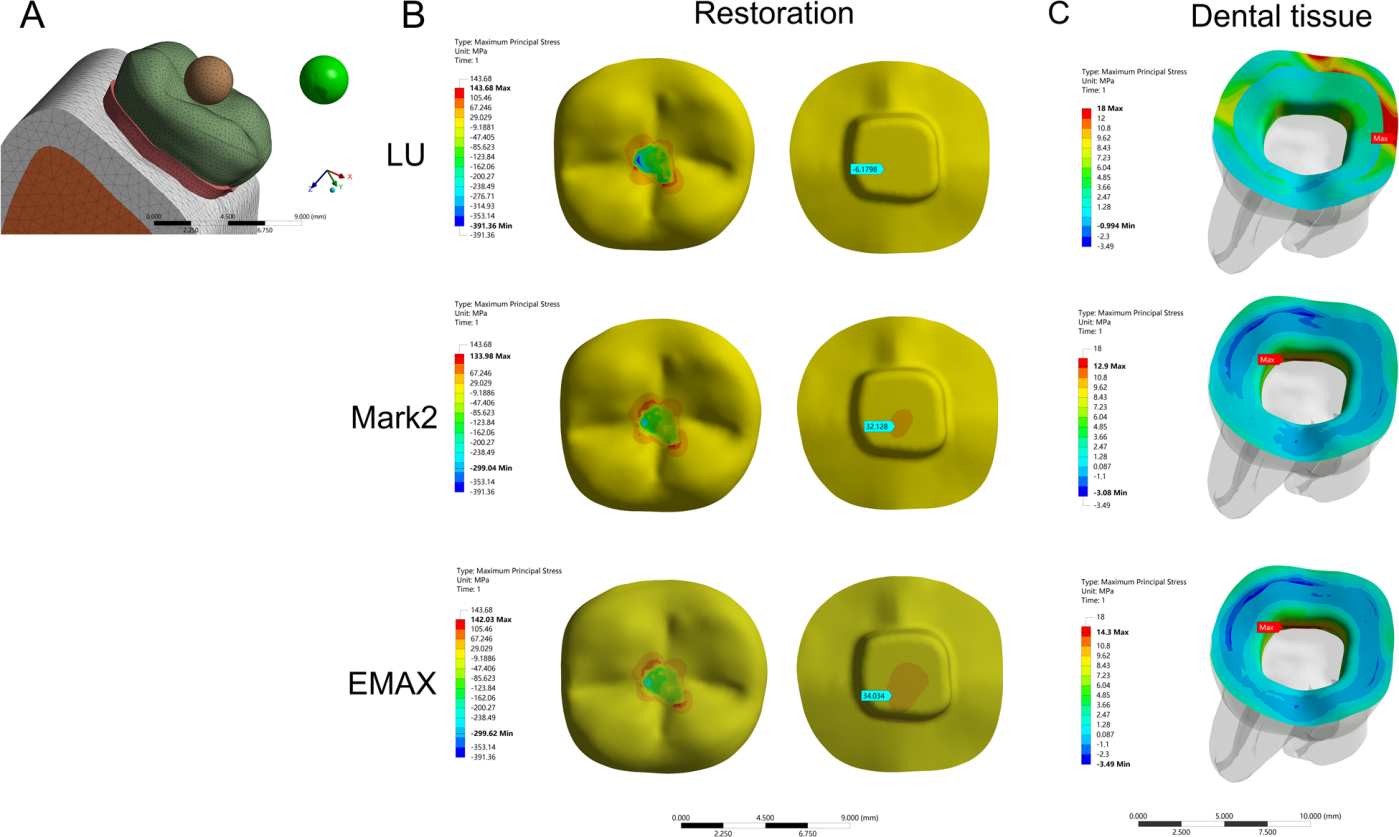
Stress distribution of the mandibular molar covered by an endocrown restoration with 1-mm thickness and a 2-mm depth of pulp chamber. A small spherical indenter (diameter: 3 mm) was assigned with Young’s modulus and Poisson’s ratio of bone and loaded at the central fossa (CF) (1.44 mm^2^) of the endocrown restorations to simulate suddenly biting a bone fragment (**A**). The restorations were made with lava ultimate (LU), feldspathic (Mark2) and lithium disilicate (EMAX). The MaxPS and MinPS values of the restoration (**B**) and tooth tissue (**C**) are displayed

**Fig. 4:**
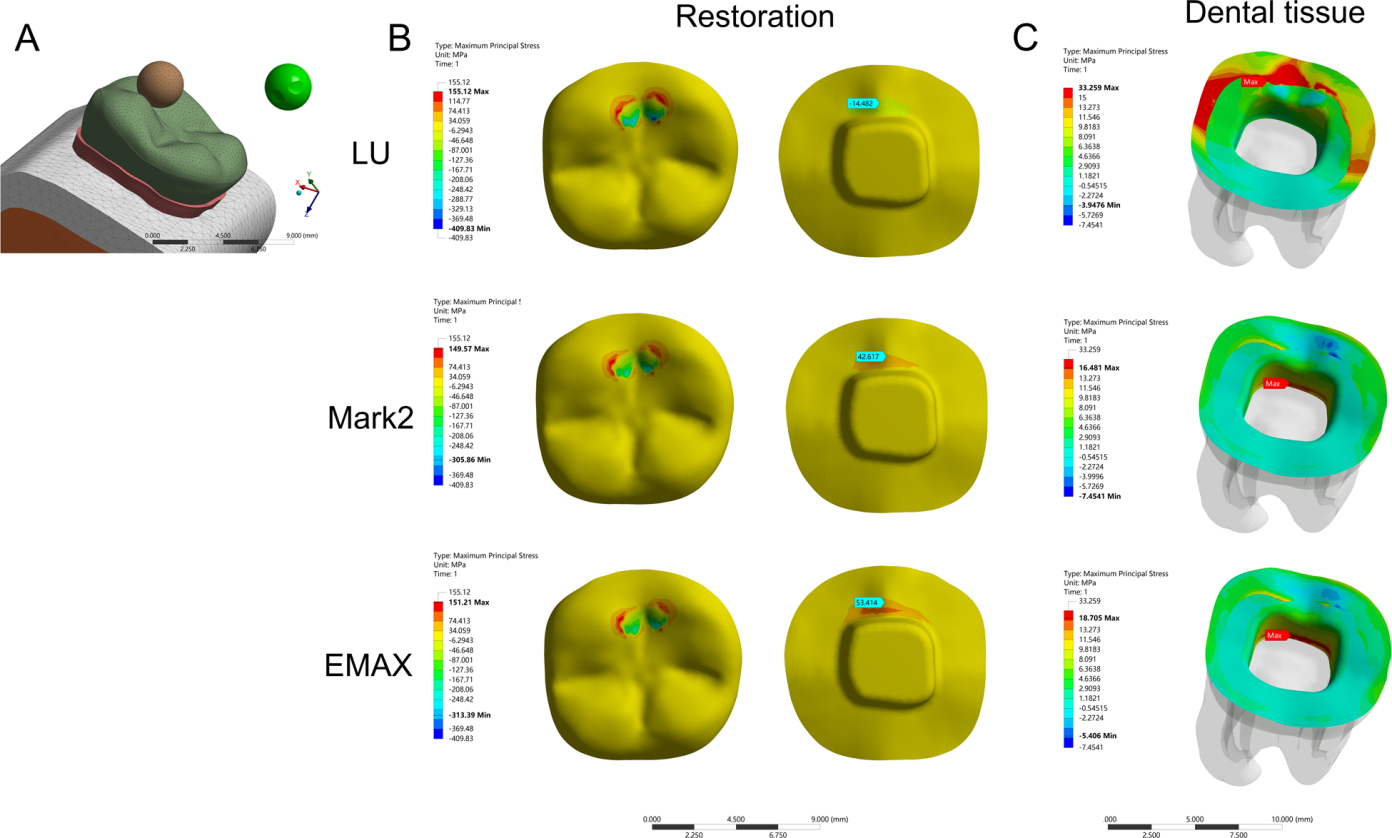
Stress distribution of the mandibular molar covered by an endocrown restoration with a 1-mm thickness and a 2-mm depth of pulp chamber. A small spherical indenter (diameter: 3 mm) was assigned with Young’s modulus and Poisson’s ratio of bone and loaded at the buccal groove (BG) (1.43 mm^2^) of the endocrown restorations to simulate suddenly biting a bone fragment (**A**). The restorations were made with lava ultimate (LU), feldspathic (Mark2) and lithium disilicate (EMAX). The MaxPS and MinPS values of the restoration (**B**) and tooth tissue (**C**) are displayed

**Fig. 5:**
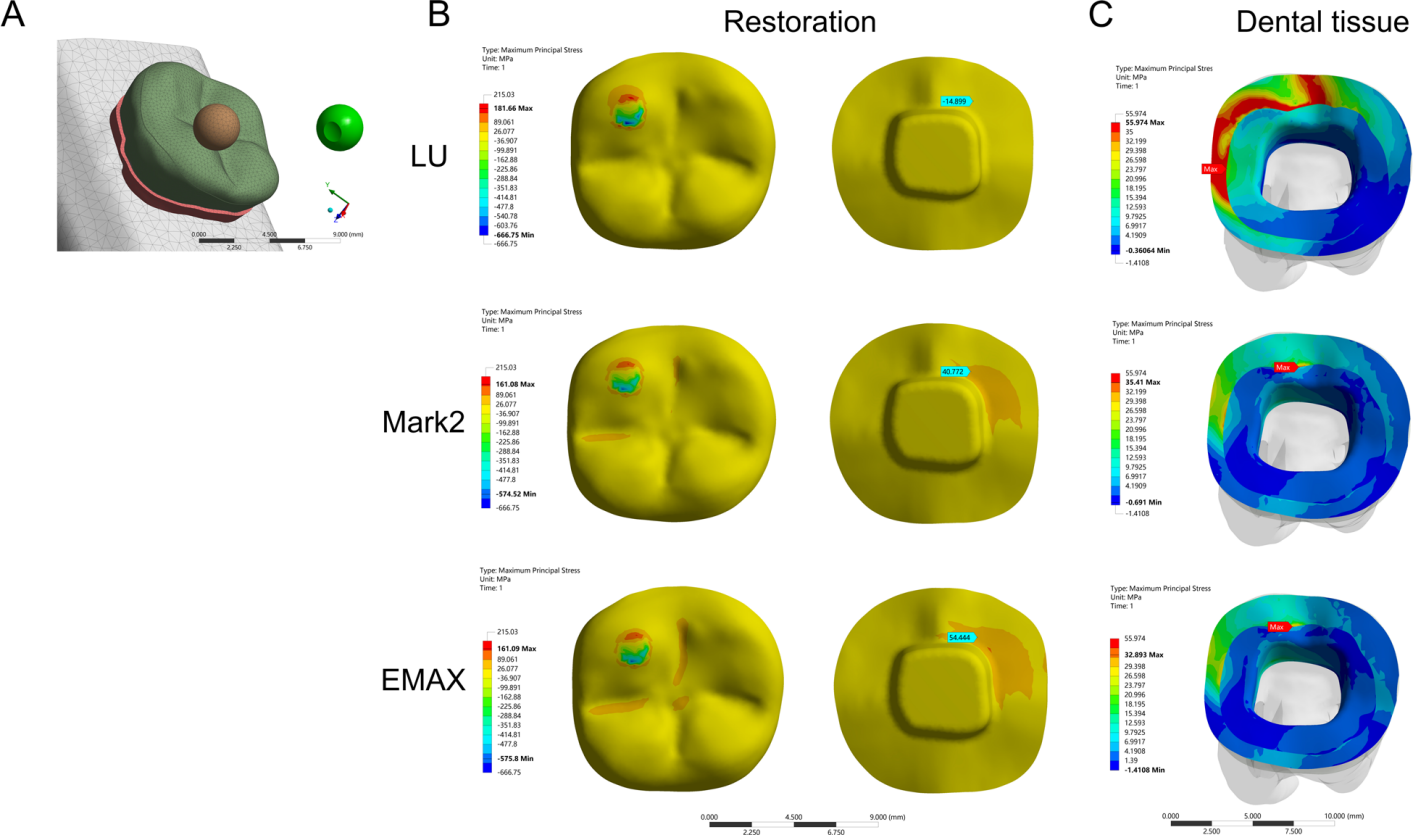
Stress distribution of the mandibular molar covered by an endocrown restoration with a 1-mm thickness and a 2-mm depth of pulp chamber. A small spherical indenter (diameter: 3 mm) was assigned with Young’s modulus and Poisson’s ratio of bone and loaded at the mesiobuccal cusp (MC) (1.43 mm^2^) of the endocrown restorations to simulate suddenly biting a bone fragment (**A**). The restorations were made with lava ultimate (LU), feldspathic (Mark2) and lithium disilicate (EMAX). The MaxPS and MinPS values of the restoration (**B**) and tooth tissue (**C**) are displayed

In the model of the endocrown restoration, physiological masticatory loads were simulated as an occlusal static load of 600 N on the *Z* axis, similar to the study by Ausiello et al. [17]. The methodology considered the contact between a food bolus (apple pulp) and the restoration surface during the closing phase of the chewing cycle. A cylinder indenter was made by subtracting the occlusal morphology of the endocrown restoration in ANSYS v18.0 software and almost covered the full occlusal (FO) surface of the endocrown restoration except for the lingual edge (108.57 mm^2^) to simulate central occlusion (Fig. 1A). A large sphere indenter (diameter: 8.57 mm) was generated by revolving in ANSYS v18.0 software and contacting the occlusal center (OC) area of the endocrown restoration (11.425 mm^2^) (Fig. 2A). Furthermore, three small sphere indenters (diameter: 3 mm) were assigned with Young’s modulus and Poisson’s ratio of bone and loaded at the central fossa (CF) (1.44 mm^2^), buccal groove (BG) (1.43 mm^2^) and mesiobuccal cusp (MC) (1.43 mm^2^) of the endocrown restoration and simulated suddenly biting bone fragments (Figs. 3A, 4A, 5A). The results in the restoration and tooth tissue were obtained using maximum principal stress (MaxPS) and minimum principal stress (MinPS) for quantitative analysis. Stress values differing by less than 5% were considered to be similar.

## Results

### Stress distribution of the endocrown restoration

In the stress analysis of the model, MaxPS denoted tensile stress (positive value), and MinPS signified compressive stress (negative value). The different loads showed different stress distributions on the endocrown restoration. The smaller the contact surface of the load, the higher MaxPS and MinPS in the five different loads. The MaxPS and MinPS units were megapascals (MPa) in the following results. In the FO load, the MaxPS (3.21) of the LU restoration was significantly lower than the MaxPS values of the Mark2 (19.5) and EMAX restorations (23.7), and the MinPS (11.9) of the LU restoration was higher than the MinPS values of the Mark2 (3.26) and EMAX (3.64) restorations (Fig. 1B). In the OC load by a large sphere, the MaxPS values of the LU (58.9), Mark2 (61.4) and EMAX (59.9) restorations were not significantly different, and the MinPS (51.2) of the LU restoration was slightly higher than the MinPS values of the Mark2 (40.3) and EMAX (41.2) restorations (Fig. 2B). In the three small sphere loads, the MaxPS and MinPS of the MC were the highest, followed by the BG and CF in the LU, Mark2, and EMAX restorations. The intaglio surface of the LU restoration showed compressive stress, whereas the intaglio surfaces of the Mark2 and EMAX restorations showed tensile stress at the site of a small sphere load (Figs. 3B, 4B, and 5B).

### Stress distribution of the tooth tissue

The FEA of tooth tissue comprised the stress analysis of enamel and dentin. Overall, the MinPS and MaxPS of the thin endocrown were significantly higher than those of tooth tissue in the five types of loads except for the LU endocrown loaded in the FO group (Table 2). In the stress distribution of tooth tissue by the FO load, the MaxPS (25.8) of tooth tissue in the LU restoration was significantly higher than those in the Mark2 (16.0) and EMAX (15.5) restorations, and the MinPS values by the three materials were not obviously different (Fig. 1C). In OC load, the stress distribution of tooth tissue showed that the MaxPS (21.7) in the LU endocrown accumulated in the external edge of enamel and was significantly higher than the MaxPS values in Mark2 (8.39) and EMAX (9.86) endocrowns, which were concentrated on the chamber wall of dentin (Fig. 2C). Similarly, stress analysis of the tooth tissue in the CF load revealed that the MaxPS (18.0) was concentrated at the external edge of enamel in the LU restoration and was significantly higher than the MaxPS values on the chamber wall of dentin in the Mark2 (12.9) and EMAX (14.3) restorations (Fig. 3C). Stress distribution by the BG load showed that the MaxPS (33.6) at the external edge of the enamel in the LU restoration was almost double those of the MaxPS values on the chamber wall of dentin in the Mark2 (16.5) and EMAX (18.7) restorations (Fig. 4C). The MaxPS values of tooth tissue in all three material restorations were at the internal edge of the enamel when the MC was loaded by the small sphere, and the MaxPS (56.0) in the LU restoration was higher than the MaxPS values in the Mark2 (35.4) and EMAX (32.9) restorations (Fig. 5C). In sum, tooth tissue received higher stress distribution in the LU endocrown restoration than in the Mark2 and EMAX restorations. The enamel supported more tensile stress, and the dentin mostly undertook the compressive stress.

**Table 2:** Stress distribution of thin endocrown restorations made with Lava Ultimate, Mark2 and EMAX (MPa)

Materials	Load site	Restoration		Tooth tissue	
		MaxPS	MinPS	MaxPS	MinPS
Lava ultimate	FO	3.21	−11.9	25.8	−1.81
	OC	58.9	−51.2	21.7	−2.2
	CF	143.68	−391.36	18	−0.99
	BG	155.12	−409.83	33.26	−3.95
	MC	181.66	−666.75	55.97	−0.36
Mark2	FO	19.5	−3.26	16	−1.55
	OC	61.4	−40.3	8.39	−3.32
	CF	133.98	−299.04	12.9	−3.08
	BG	149.57	−305.86	16.48	−7.45
	MC	161.08	−574.52	35.41	−0.69
EMAX	FO	23.7	−3.64	15.5	−1.55
	OC	59.9	−41.2	9.86	−3.13
	CF	142.03	−299.62	14.3	−3.49
	BG	151.21	−313.39	18.71	−5.41
	MC	161.09	−575.8	32.89	−1.41

*MaxPS* maximum principal stress, *MinPS* minimum principal stress

A positive value denoted tensile stress, and a negative value signified compressive stress. A total of 600 N was vertically loaded on an endocrown restoration with 1 mm of thickness and 2 mm of intracoronal extension by the five methods. FO: indenter loading on the full occlusal surface except for the lingual edge; OC: indenter loading on the occlusal center of restoration. Moreover, a small sphere indenter was loaded at the central fossa (CF), buccal groove (BG), and mesiobuccal cusp (MC) of the coronal restorations

## Discussion

Endocrown has been considered a reliable alternative in the restoration of teeth with bad damage after root canal treatment [4, 13]. From minimally invasive dentistry, conservative treatment in tooth preparation for an endocrown is inevitable to decrease the thickness of the restoration. Furthermore, when the height of the crown is limited, whether a thin endocrown restoration meets the clinical requirement still needs further investigation. Finite element analysis has often been used in dental biomechanical research to estimate the stress distribution in a dental field and to predict the practicability of the restoration. FEA is a reproducible and noninvasive technique that simulates the oral environment and obtains stress values at any point [18–21]. In this study, we constructed a model of endocrown restoration with a pulp chamber thickness of 1 mm and a depth of 2 mm by FEA and evaluated the stress distribution of the restoration made by LU, Mark2, and EMAX materials. Thin restorations were considered to accumulate more stress in the structures than thick restorations and may induce the formation of cracks and increase the failure risk in critical thickness restorations [14, 22, 23]. Therefore, 1 mm thickness of endocrown was selected as a critical thickness restoration to analyze the stress distribution. In addition, a 2-mm depth of the pulp chamber was considered optimal in endocrown restoration [24, 25].

In this study, three endocrown restorations under five different loads were analyzed. The indenter contacting the almost FO surface and the OC of the restoration, respectively, denotes a situation of central occlusive and biting food. The three sites of restoration (CF, BG, and MC) were loaded by a small spherical indenter intended to simulate bone fragments. In stress analysis, MaxPS is a tensile stress measurement used to judge the material failure that is assumed to be due to brittleness and implies a fracture tendency of the components [26]. In the larger contact area of load (FO), the LU restoration showed a lower MaxPS than the Mark2 and EMAX restorations, whereas the smaller contact area of load (CF, BG, and MC) revealed a higher stress concentration in the LU restoration than in the Mark2 and EMAX restorations. This phenomenon indicated that the LU material dispersed the stress at the evenly occluded contact, but high tensile stress occurred in the highlighted site of the LU restoration when encountering an unexpected bite. Furthermore, regardless of whether there was a large or small contact area of restoration in the test loads, the tooth tissue sustained significantly higher tensile stress with the LU restoration than with the Mark2 and EMAX restorations, and the enamel received more tensile stress than the dentin. The elastic modulus of the LU restoration was significantly lower than those of the Mark2 and EMAX restorations [11], and the low elastic modulus of the materials transferred more stress to dental tissue, which is consistent with the Yamanel et al.’s study [27]. Thus, the high elastic modulus of the materials may absorb more stress and reduce the stress distribution of tooth tissue. The low elastic modulus of the LU restoration mainly showed compressive stress in the whole restoration in the five test loads and transferred stress to the tooth tissue. The high elastic modulus of the Mark2 and EMAX restorations displayed little tensile stress on the intaglio surface of the restoration, and less stress was passed to tooth tissue. The data suggested that a thin Mark2 or EMAX restoration may tend to develop radial cracks and protect the tooth tissue.

The effect of material thickness on restoration fractures has been studied by some authors [14, 16, 22, 28, 29]. Thinner restorations accumulate more stress in the structures than thicker restorations and may cause the formation of cracks and increase the fracture risk in critical thickness. In our study, almost the same contact area of the indenter was loaded on the CF, BG, and MC of the restoration. We found that the tensile stress levels of the intaglio surfaces of the Mark2 and EMAX restorations in the BG and MC groups were significantly higher than those in the CF group. The thickness of the CF was obviously greater than those of the BG and the MC in endocrown restorations due to the 2-mm depth of the intracoronal extension. Furthermore, despite the lack of a significant difference between the tensile stress of the intaglio surfaces of the Mark2 or EMAX restorations in the BG and MC groups, the MaxPS was concentrated on the chamber wall of the tooth tissue in the BG group, and MaxPS occurred on the enamel in the MC group. Meanwhile, the Mark2 and EMAX restorations in the CF group transferred the MaxPS to the chamber wall of the tooth tissue. The results indicated that the different sites of load on the occlusal surface of the endocrown may result in the different stress distributions of tooth tissue. A lower MaxPS tended to concentrate on the dentin, and a higher MaxPS appeared on the enamel. The thin endocrown restoration sustained primary stress, whereas the tooth tissue received relatively low stress when encountering an unexpected bite, such as a bone fragment or small stone.

In the full-coverage occlusal of load, tooth tissue sustained the higher tensile stress than the LU endocrown restoration, and inverse results in Mark2 and EMAX restoration, which indicated that the low elastic modulus of materials may take little tensile stress, and the primary stress was transferred to the tooth tissue in the central occlusion. A high elastic modulus material will sustain a larger amount of stress and reduce the tensile stress on the tooth tissue. When 600 N was vertically loaded on the OC area of the thin endocrown, the MaxPS on the tooth tissue became lower than that in the LU restoration, and the MaxPS was transferred on the chamber wall in the Mark2 and EMAX restorations. This outcome might be related to the thickness of the restoration. The central thickness of the endocrown was three times that of the surroundings. Therefore, during rehabilitation of the thin endocrown, intracoronal extension is helpful to disperse the stress of the tooth tissue. In our FEA study, only a 2-mm depth was employed. We still need to explore the effects of other depths of extension on the stress distribution of tooth tissue with thin endocrown.

## Conclusion

Within the limitations of this FEA study of thin endocrown restoration of an endodontically treated molar, the following conclusions were drawn:

1. The LU endocrown transferred more stress to tooth tissue than the Mark2 and EMAX restorations, irrespective of the loads.
2. The smaller the contact surface when the restoration was loaded, the higher MaxPS and MinPS were in the three material restorations.
3. At the same load force and area, the MaxPS on the endocrown restoration and tooth tissue at the MC load was higher than that at the CF and the BG, regardless of the restorative materials.

## Supplementary information

Supplementary Material
