# Supplementary material for: Resistance fracture of minimally prepared endocrowns made by three types of restorative materials: a 3D finite element analysis
Source: J Mater Sci Mater Med. 2021 Oct 30;32(11):137. doi: 10.1007/s10856-021-06610-x (PMC8557147; doi:10.1007/s10856-021-06610-x)
Supplement: Supplementary file 1 — Supplementary Material [file 10856_2021_6610_MOESM1_ESM.pdf]

ELSEVIER

Certificate of Elsevier Language Editing Services

The following article was edited by Elsevier Language Editing Services:

"The resistance fracture of minimally prepared endocrown made by the three types of restorative materials: a 3D Finite Element Analysis"

Authored by:

Zhongchun Tong

Date: 30-Jun-2021

Serial number: LE-215259-C64B9C041938
